# Rivaroxaban plus aspirin vs. dual antiplatelet therapy in endovascular treatment in peripheral artery disease and analysis of medication utilization of different lesioned vascular regions

**DOI:** 10.3389/fsurg.2023.1285553

**Published:** 2023-11-09

**Authors:** SiYan Huo, Jun Cheng

**Affiliations:** Department of Vascular Surgery, The First Affiliated Hospital of Chongqing Medical University, Chongqing, China

**Keywords:** peripheral arterial disease (PAD), under-the-knee artery disease, rivaroxaban, aspirin, endovascular treatment (EVT), bleeding risk, atherosclerosis

## Abstract

**Background:**

In the management of Peripheral Arterial Disease (PAD), the administration of anticoagulant or antiplatelet agents is imperative. The use of Dual Antiplatelet Therapy (DAPT) in conjunction with rivaroxaban has shown potential in mitigating adverse outcomes. Given the heterogeneity in the pathology of lower limb arteries, there is a compelling case for individualized treatment strategies.

**Methods:**

In a single-center retrospective study on pharmacotherapy for peripheral artery disease, patients were treated with either aspirin combined with rivaroxaban or aspirin coupled with clopidogrel. The primary efficacy outcome encompassed a composite of increases in the Rutherford classification, acute limb ischemia, amputations due to vascular causes, target lesion revascularization, myocardial infarction, ischemic stroke, and cardiovascular death. The primary safety outcome was major bleeding, as defined by the Thrombolysis in Myocardial Infarction (TIMI) criteria; meanwhile, major bleeding as categorized by the International Society on Thrombosis and Haemostasis (ISTH) served as a secondary safety outcome. The study differentiated between two subgroups: patients with only above-the-knee and below-the-knee arterial lesions.

**Results:**

From January 2016 to December 2021, 455 patients received either clopidogrel plus aspirin or rivaroxaban plus aspirin following endovascular treatment (EVT). The rivaroxaban group (*n* = 220) exhibited a lower incidence of primary efficacy outcomes [49.1% vs. 60.4%, hazard ratio (HR) 0.77, *P* = 0.006] but had more TIMI major bleeding events (5.9% vs. 2.1%, HR 2.6, *P* = 0.04). ISTH major bleeding events did not show a significant difference, though a higher percentage of rivaroxaban patients discontinued medication due to bleeding (10% vs. 4.7%, HR 2.2, *P* = 0.03). In the above-the-knee arterial disease subgroup, the rivaroxaban group demonstrated a lower incidence of primary efficacy outcomes (28.2% vs. 45.2%, HR 0.55, *P* = 0.02). In the below-the-knee arterial disease subgroup, no significant difference was observed in the occurrence of primary efficacy events between the two groups (58.7% vs. 64.8%, HR 0.76, *P* = 0.14).

**Conclusion:**

Rivaroxaban plus aspirin improved outcomes compared to DAPT in patients with lower extremity artery disease. Similar findings were observed in the above-the-knee artery lesion-only group. However, in the below-the-knee artery lesion-only group, rivaroxaban plus aspirin did not surpass DAPT in efficacy. Regarding safety, rivaroxaban plus aspirin exhibited higher bleeding risks and more frequent treatment discontinuation than aspirin combined with clopidogrel.

## Introduction

Peripheral arterial disease (PAD) is traditionally viewed as an outcome of systemic atherosclerosis, manifesting through cardiovascular symptoms and events related to limb involvement. However, emerging evidence underscores the unique attributes of PAD, distinguishing it from coronary and cerebral artery diseases. Specifically, PAD is associated with an elevated risk of adverse limb events, encompassing acute limb ischemia and amputation, in addition to significant cardiovascular events. Such outcomes frequently correlate with distinctive high-risk factors intrinsic to PAD (1, 2). Peripheral arterial disease manifests with a spectrum of symptoms, ranging from intermittent claudication, which can impede daily activities, to more severe limb-threatening ischemia necessitating revascularization to avert or mitigate tissue necrosis (3, 4), although surgical interventions prove efficacious for specific ailments, accumulated clinical experience suggests that surgery alone doesn't assure enduring favorable outcomes. Consequently, prolonged administration of anticoagulant or antiplatelet medications might be indispensable to preserving such outcomes over extended periods (5–9). For an extended duration, antiplatelet therapy has been esteemed as the cornerstone treatment for both acute and chronic afflictions of the coronary and peripheral arteries (10–12), recent studies have elucidated that dual antiplatelet therapy, encompassing acetylsalicylic acid and clopidogrel, results in a reduced prevalence of thrombotic events among patients with acute coronary syndromes and those undergoing percutaneous coronary interventions, in comparison to monotherapy with acetylsalicylic acid (13, 14).This insight prompted a paradigmatic shift in treatment approaches. Yet, ensuing research indicates that, even when treated with the most potent P2Y12 inhibitors, approximately one in ten patients undergoes recurrent thrombotic episodes within the initial year following an acute coronary syndrome event (15).This suggests that other mechanisms beyond platelet function may contribute to thrombosis development (13, 16, 17), questions still remain regarding the most effective antithrombotic therapy for the long-term management of chronic vascular diseases. While dual antiplatelet therapy is frequently employed in peripheral arterial disease, its backing is predominantly from observational studies or extrapolations from trials in coronary artery disease. These trials primarily emphasize cardiovascular outcomes and stent thrombosis rather than the prognosis related to limb health (4, 12, 17). In clinical settings, the incidence of stenosis post-percutaneous angioplasty in the femoral-popliteal region, under standard treatment with dual antiplatelet therapy, ranges between 17% and upward of 40%. Notably, the likelihood of stenosis escalates in proportion to the lesion's length (18–20). The treatment effect is significantly unsatisfactory.

Rivaroxaban is a type of selective factor Xa inhibitor used to prevent and treat venous thrombosis, as well as prevent stroke and thromboembolism in individuals with atrial fibrillation (21, 22). In the COMPASS trial, encompassing a substantial cohort of patients post-lower extremity revascularization, nearly 20% of the placebo group encountered a primary composite outcome—this included acute limb ischemia, major vascular-driven amputation, myocardial infarction, ischemic stroke, or cardiovascular-related mortality—within a span of three years. Intriguingly, supplementing aspirin (100 mg daily) with rivaroxaban (2.5 mg twice daily) diminished the likelihood of such outcomes by an estimated 15% (23). The regimen combining rivaroxaban, administered at 2.5 mg twice daily, with aspirin has garnered endorsement from numerous international organizations. Consequently, its adoption is on the rise and is progressively being incorporated into prevailing international guidelines (11, 12).

While antiplatelet therapy has been recognized as a pivotal treatment for conditions in both coronary and peripheral arteries, the efficacy of dual antiplatelet therapy, particularly the combination of acetylsalicylic acid and clopidogrel, has been substantiated in reducing thrombotic events in specific cardiovascular conditions. Yet, for PAD, the most effective long-term anticoagulant strategy remains ambiguous, especially when considering the variations in vascular segments. For instance, femoral and popliteal arterial lesions predominantly demonstrate a significant atheromatous plaque presence, whereas below-the-knee lesions are more characterized by thrombotic manifestations with less atherosclerotic involvement (24, 25). This heterogeneity in pathology raises questions about the appropriateness of a generalized postoperative therapeutic approach for all PAD cases. This study is designed to critically evaluate and compare the efficacy and safety of a combination of rivaroxaban and aspirin vs. that of clopidogrel and aspirin in PAD patients. Specifically, it seeks to discern whether the location of vascular lesions influences therapeutic outcomes. By addressing this gap, the research aspires to provide the scientific community with robust and evidence-based guidance on the optimal therapeutic strategies for PAD.

## Methods

### Study design

In this single-center retrospective study, the inclusion criteria encompassed: individuals aged over 40; documented clinical evidence of percutaneous transluminal angioplasty for atherosclerotic ischemia in the lower extremity, manifesting symptoms such as ambulatory limitation, intermittent claudication, and ischemic ulceration; imaging confirmation of luminal constriction from the iliac to the distal artery within the preceding year; a record of successful angioplasty devoid of procedural setbacks; a preoperative Rutherford classification confined to grades 2–5; target lesion segment stenosis exceeding 70% or presenting as an occlusive lesion; and an absence of a history regarding lower extremity arterial revascularization. Exclusion criteria incorporated: asymptomatic subjects or those presenting negligible intermittent claudication; preoperative extensive ischemic necrosis in the concerned limb; utilization of antiplatelet agents other than clopidogrel or aspirin, or anticoagulants other than rivaroxaban; known adverse reactions or allergies to P2Y12 inhibitors; severe hepatic ailments compromising the anticoagulation pathway and increasing bleeding risks; renal insufficiency marked by a creatinine clearance under 30 ml/min; intraoperative angiography indicating distal vascular embolism; and a projected life expectancy below 5 years.

The study delineated both primary and secondary endpoints. Primary endpoints encompassed: a rise in the Rutherford classification by a minimum of one grade relative to the post-angioplasty status, acute limb ischemia, amputation due to vascular causes, target lesion revascularization, myocardial infarction, ischemic stroke, and cardiovascular death. Secondary endpoints included: an elevation in the Rutherford classification by at least one grade post-angioplasty, acute limb ischemia, vascular-motivated amputation, target lesion revascularization, myocardial infarction, ischemic stroke, cardiovascular-related death, fatal heart disease, thrombotic events in the coronary or peripheral arteries, and revascularization requirements in the coronary or peripheral arteries.

In Table 1, the “primary efficacy outcomes” and “secondary efficacy outcomes” represent the composite outcome events for the primary endpoints and secondary endpoints, respectively. To more precisely determine which events impact overall therapeutic efficacy, we analyzed individual outcomes from our primary efficacy outcomes post-EVT. Recognizing atherosclerosis as a systemic condition, we crafted secondary endpoints that encompass wider cardiovascular adversities. Our “key secondary efficacy outcomes” focus solely on adverse lower limb events, while “key other secondary efficacy outcomes” emphasize only cardiovascular events.

**Table 1:** Efficacy outcomes.^a^

Outcome	Rivaroxaban (*N* = 220) Patients with event No. %	DAPT (*N* = 235) Patient with event No. %	Hazard ratio (95% CI)	*P* value
Primary efficacy outcome: increase in Rutherford classification by at least 1 grade over post-angioplasty, acute limb ischemia, amputation for vascular reasons, target lesion revascularization, myocardial infarction, ischemic stroke, death from cardiovascular cause	108 (49.1%)	142 (60.4%)	0.77 (0.60, 0.99)	0.006
Increase in Rutherford classification by at least 1 grade over post-angioplasty	78 (35.5)	106 (45.1)	0.72 (0.54, 0.97)	0.03
Acute limb ischemia	13 (5.9)	24 (10.2)	0.58 (0.30, 1.14)	0.11
Amputation for vascular reasons	24 (10.9)	38 (16.2)	0.73 (0.44, 1.20)	0.12
Target lesion revascularization	37 (16.8)	65 (27.3)	0.57 (0.38, 0.86)	0.007
Myocardial infarction	7 (3.2)	9 (3.8)	0.84 (0.31, 2.25)	0.73
Ischemic stroke	8 (3.6)	15 (6.4)	0.57 (0.24, 1.34)	0.2
Death from cardiovascular causes	2 (0.9)	4 (1.7)	0.54 (0.10, 2.96)	0.48
Secondary efficacy outcome: increase in Rutherford classification by at least 1 grade over post-angioplasty, acute limb ischemia, amputation for vascular reasons, target lesion revascularization, myocardial infarction, ischemic stroke, death from cardiovascular causes, fatal heart disease, coronary or peripheral thrombosis events, coronary or peripheral artery requiring revascularization	122 (55.5)	156 (66.4)	0.73 (0.58, 0.93)	0.01
Key secondary efficacy outcome: increase in Rutherford classification by at least 1 grade over post-angioplasty, acute limb ischemia, amputation for vascular reasons, target lesion revascularization	105 (47.7)	132 (56.2)	0.79 (0.61, 1.02)	0.076
Key other secondary efficacy outcome: myocardial infarction, ischemic stroke, death from cardiovascular causes, fatal heart disease, coronary or peripheral thrombosis events, coronary or peripheral artery requiring revascularization	18 (8.2)	29 (12.3)	0.65 (0.36, 1.18)	0.16
Coronary or peripheral artery requiring revascularization	83 (37.7)	108 (46.0)	0.77 (0.58, 1.03)	0.082
Coronary or peripheral arterial thrombotic events	9 (4.1)	17 (7.2)	0.56 (0.25, 1.25)	0.56
Subgroup of suprapopliteal primary efficacy outcome	22 (28.2)	38 (45.2)	0.54 (0.32, 0.92)	0.024
Subgroup of suprapopliteal secondary efficacy outcome	27 (35.1)	47 (55.3)	0.56 (0.35, 0.90)	0.017
Subgroup of infrapopliteal primary efficacy outcome	54 (58.7)	59 (64.8)	0.75 (0.52, 1.09)	0.13
Subgroup of infrapopliteal secondary efficacy outcome	62 (66.0)	65 (71.4)	0.79 (0.56, 1.12)	0.19

^a^ Event rates are provided for the adjudicated events that include the primary efficacy outcomes, secondary efficacy outcomes, and their respective components. CI denotes confidence interval.

Patients in the rivaroxaban group were prescribed a regimen of 5 mg rivaroxaban twice daily combined with 100 mg aspirin once daily. In contrast, the aspirin group followed a dual-antiplatelet strategy, receiving 75 mg clopidogrel once daily along with 100 mg aspirin once daily.

The primary safety endpoint of the study was major bleeding based on the TIMI criteria, while the secondary safety endpoint was bleeding events following the criteria set by the ISTH (26, 27).

Clinical data were gleaned retrospectively from inpatient medical documentation. Preoperatively, patients were administered 4,000 units of low molecular weight heparin subcutaneously twice daily, barring any contraindications. During the surgical procedure, patients were afforded local anesthesia, intravenous sedation, and analgesia, complemented by systemic heparinization. The femoral artery puncture approach was determined by the target lesion's location, employing suitable guidewires and catheters. Balloon angioplasty and stenting were undertaken as necessitated. Post-surgical prescriptions typically included either rivaroxaban (5 mg twice daily) coupled with aspirin (100 mg daily) or clopidogrel (75 mg daily) in conjunction with aspirin (100 mg daily), administered orally. A survival analysis was employed to discern variations in outcomes and delve into contributing determinants. Adherence to the prescribed medication regimen was closely monitored for a minimum duration of 1 year.

To elucidate whether the location of vascular lesions impacts therapeutic outcomes, we segregated patients from the overall cohort into two specific categories: those with exclusive above-the-knee artery lesions and those with exclusive under-the-knee artery lesions. Disease Limited to above-the-knee artery denotes above-the-knee stenosis (>50%) requires endovascular therapy. Under this condition, under-the-knee stenosis with maintained luminal patency does not require EVT, preserving distal blood flow. Disease Limited to under-the-knee artery encompasses arterial stenosis or occlusion that results in interrupted distal limb blood flow, requiring endovascular therapy (EVT). Under this condition, above-the-knee artery disease typically presents with stenosis degrees below 50%. Consequently, this delineation yielded two distinct subgroups: one subgroup with isolated above-the-knee artery lesions treated with rivaroxaban and another treated with dual antiplatelet therapy; and a second subgroup with isolated under-the-knee artery lesions treated similarly. Patients with lesions involving the entire lower limb artery, such that both the above-knee and below-knee arteries require endovascular therapy (EVT), were not included in the subgroup analysis. The statistical methodologies employed for these subgroups mirrored those utilized for the broader patient sample.

In this study, prospective patient follow-up, data collection, and organization were executed with the explicit consent of participants and had the endorsement of the Institutional Review Board at the First Affiliated Hospital of Chongqing Medical University. The stewardship of data integrity and the safety of participants were conferred upon an independent Data Monitoring Committee, comprising scholars with no affiliations to the study's sponsors. Furthermore, a Clinical Events Committee, staffed by seasoned clinicians from the First Affiliated Hospital of Chongqing Medical University, carried out blinded evaluations of bleeding events and clinical outcomes, ensuring an impartial assessment. Despite the study's classification as a low-risk retrospective cohort, in adherence to the stipulations set by the Ethics Committee of the First Affiliated Hospital of Chongqing Medical University, an informed consent form was crafted for patients. It was confirmed that all participants, or their legal guardians, provided informed consent. This research adhered to the principles of the Declaration of Helsinki and incorporated the examination of anonymized data.

### Statistic analysis

SPSS 26.0 software was utilized for statistical analysis. Continuous variables were assessed using the Kaplan–Meier (K-M) analysis. Normally distributed data were expressed as mean (±standard deviation) and analyzed with independent samples *t*-test for between-group comparisons, and paired *t*-test for within-group comparisons. Non-normally distributed data were presented as median (P25, P75) and compared using Mann–Whitney *U* test. Categorical variables were reported as *n* (%) and analyzed using chi-square test or Fisher's exact test. K-M analysis evaluated 1-year postoperative patency, while Cox proportional hazards model assessed influencing factors and estimated hazard ratios with 95% confidence intervals. All *p*-values were two-sided and derived from log-rank test, with significance set at *p* < 0.05.

## Result

### Patients

**Figure 1:**
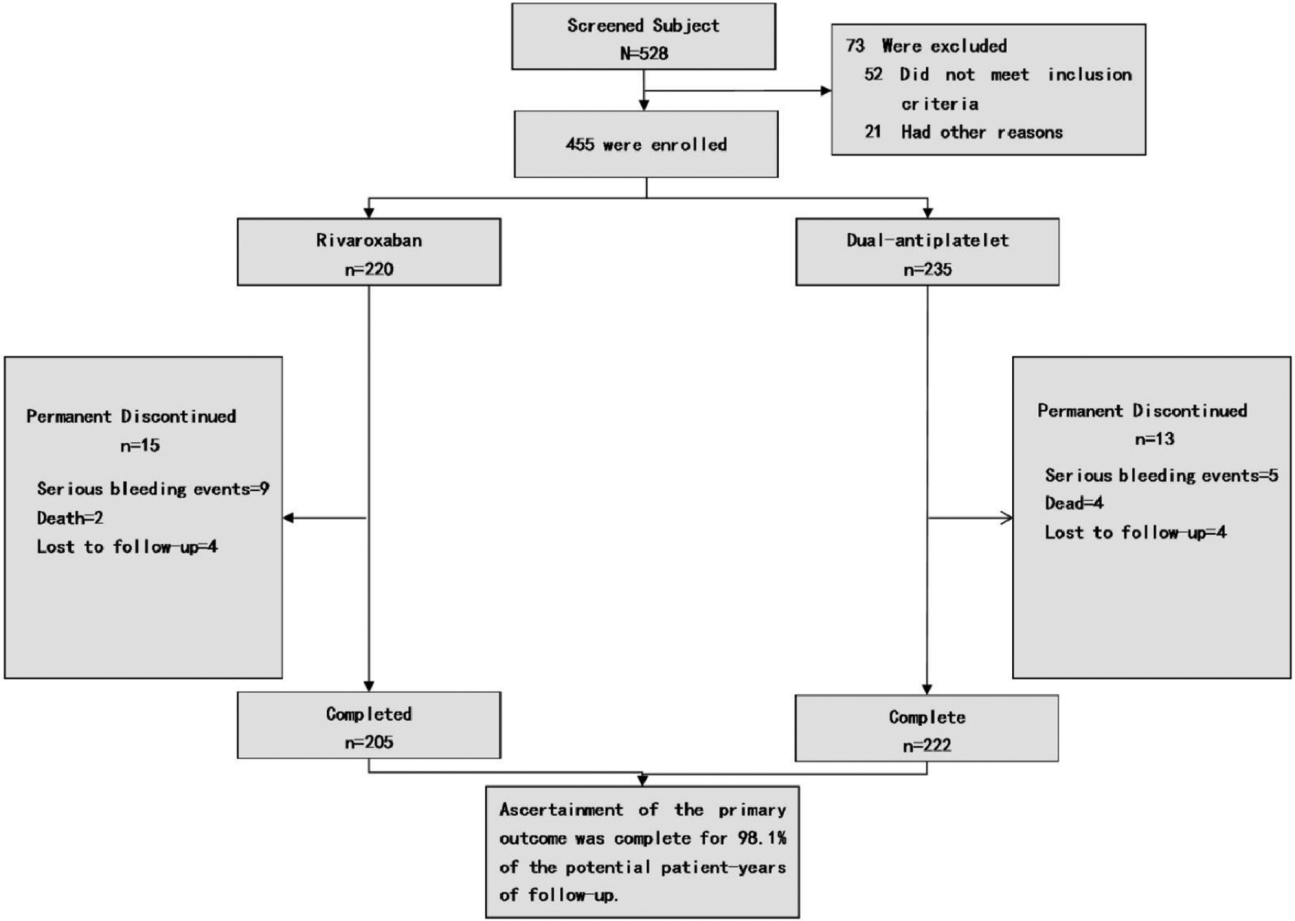
Enrollment and outcomes. Patients may be excluded from the study due to various factors. The category of “Other reasons” for exclusion encompasses patient non-consent, potential non-adherence, subject's recall ambiguity regarding treatment processes and disease progression, as well as the principal investigator's decision to discontinue the patient's participation in the trial.

From the 528 symptomatic PAD patients (Rutherford categories 2–5) screened for study eligibility, 73 were deemed ineligible based on the set criteria. The study thus encompassed 455 qualifying patients (comprising 127 women and 328 men; average age: 74.5 ± 9.3 years, age range: 44–95 years). Of these, 220 were administered rivaroxaban combined with aspirin, while 235 were prescribed clopidogrel in conjunction with aspirin as postoperative medication, as delineated in Figure 1. Data were accrued bi-monthly over a year-long follow-up duration. Baseline attributes were consistently distributed across both primary groups and their respective subgroups. Pre-surgery symptoms manifested as intermittent claudication in 60.8%, resting pain in 20.8%, and minor tissue loss in 18.4% of patients. Recorded comorbidities included diabetes (48.1%), hypertension (59.1%), coronary artery disease (29%), and ACEI/ARB medication utilization (58.5%). On admission, hyperlipidemia was identified in 65.3% of patients, with 65.1% on statin therapy. Notably, 57.8% had a smoking history prior to admission. Comprehensive baseline characteristics are detailed in Table 2.

**Table 2:** Baseline characteristics of the patients.^a^

Variable	Rivaroxaban	DAPT	*P*
Median age (IQR)—year	77	76	0.40
Female sex-no. (%)	53 (24.1)	51 (21.7)	0.54
Median body-mass index(IQR)^b^	25.6 (21.8, 29.3)	25.6 (22.3, 28.9)	0.90
Diabetes-no. (%)	106 (48.2)	113 (48.1)	0.98
Smoking-no. (%)			0.67
Current	128 (58.2)	134 (57.0)	
Former	18 (8.2)	25 (10.6)	
Never	74 (33.6)	76 (32.3)	
Alcohol use-no. (%)			0.16
Never	129 (58.6)	117 (49.8)	
Rarely	34 (15.5)	42 (17.9)	
Currently consumes	57 (25.9)	76 (32.3)	
Baseline CrCl, mg/dl-no. (%)			0.27
≤50	9 (4.0)	20 (3.8)	
>50 and <80	91 (41.4)	97 (41.3)	
≥80	120 (54.5)	118 (50.2)	
>95	41 (18.6)	43 (18.3)	
Hypertension-no. (%)	127 (57.7)	142 (60.4)	0.56
Cardiovascular disease-no. (%)^c^	60 (27.3)	72 (30.6)	0.43
Carotid artery disease-no. (%)	59 (26.8)	72 (30.6)	0.37
Hyperlipidemia-no. (%)	142 (64.5)	155 (66.0)	0.75
Cholesterol, mmol/l(IQR)	4.3 (3.1, 5.5)	4.5 (3.5, 5.6)	0.15
HDL cholesterol, mmol/l(IQR)	1.4 (0.5, 2.3)	1.4 (0.9, 1.9)	0.36
LDL cholesterol, mmol/l(IQR)	2.5 (1.0, 4.0)	2.5 (1.3, 3.7)	0.58
Rutherford category-no. (%)			0.90
2	2 (0.9)	3 (0.7)	
3	130 (59.1)	274 (60.1)	
4	43 (19.5)	95 (20.8)	
5	45 (20.5)	84 (18.4)	
Lesion location-no. (%)			0.62
Limited to above-the-knee artery	78 (35.9)	84 (35.9)	
Limited to under-the-knee artery	92 (42.4)	91 (38.9)	
Entire lower extremity arterial	47 (21.7)	59 (25.2)	
Lesion length, cm(IQR)	21.4 (8.0, 34.8)	23.5 (9.5, 37.5)	0.13
Lesion severity-no. (%)			0.16
Stenosis	87 (39.5)	78 (33.2)	
Occlusion	133 (60.5)	157 (66.8)	
Preoperative ABI(IQR)	0.30 (0.16, 0.44)	0.28 (0.16, 0.40)	0.37
Postoperative ABI(IQR)	0.78 (0.69, 0.87)	0.77 (0.68, 0.86)	0.79
Preoperative TBI(IQR)	0.19 (0.14, 0.25)	0.20 (0.15, 0.25)	0.11
Postoperative TBI(IQR)	0.70 (0.64, 0.74)	0.70 (0.65, 0.77)	0.22
Stent placement-no. (%)			0.17
Bare metal	57 (25.9)	45 (19.1)	
Drug-eluting	49 (22.3)	64 (27.2)	
Balloon placement-no. (%)			0.75
Bare	92 (41.8)	90 (38.3)	
Drug-coated	120 (54.5)	136 (57.9)	
Residual stenosis(IQR)	13 (7, 19)	13 (9, 17)	0.42
History of vascular angioplasty-no. (%)	43 (19.5)	48 (21.1)	0.69
Statin-no. (%)	142 (64.5)	154 (65.5)	0.82
ACE inhibitor or ARB-no. (%)	126 (57.3)	140 (59.6)	0.62

^a^ There were no significant differences between groups. Percentages may not total 100 because of rounding. Continuous data are presented as the means ± standard deviation (if it matches normal distribution) or Median (interquartile range) (if it matches skewed distribution); categorical data are given as the counts (percentage). Percentages were based on the number of subjects in the column heading as the denominator unless specified otherwise. ACE denotes angiotensin-converting enzyme; ARB angiotensin-receptor blocker, ABI, ankle-brachial index; TBI, toe-brachial index; CrCl, creatinine clearance; HDL, high-density lipoprotein; LDL, low-density cholesterol; IQR denotes interquartile range.

^b^ The body-mass index is the weight in kilograms divided by the square of the height in meters.

^c^ Cardiovascular diseases is defined as myocardial infarction, percutaneous coronary intervention, or coronary­artery bypass grafting (CABG).

### Efficacy endpoints

#### Primary endpoints

**Figure 2:**
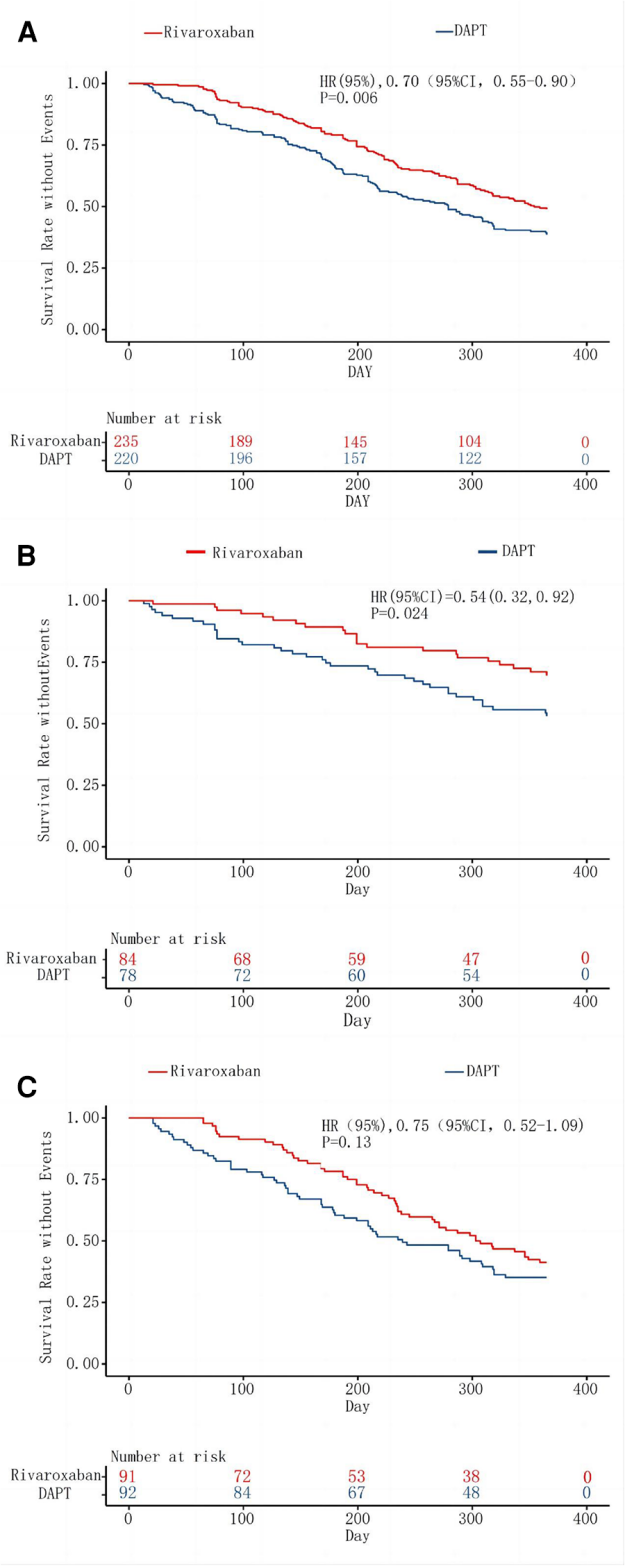
Kaplan–Meier analysis of the primary composite efficacy outcome. The inset shows the same data on an expanded y axis. Kaplan–Meier curve A assesses survival rate without Events for the primary composite efficacy outcome in lower extremity disease, regardless of above-the-knee or under-the-knee involvement. Curve B focuses on isolated above-the-knee artery disease, while Curve C examines isolated under-the-knee artery disease, both for the primary composite efficacy outcome's event-free survival rate.

**Figure 3:**
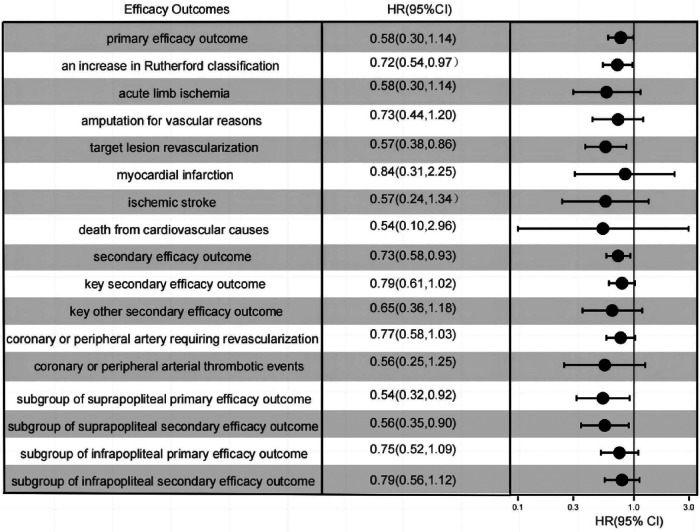
One-year event rates of all efficacy outcomes, as well as the one-year event rates of the primary efficacy composite outcomes within the subgroups of isolated suprapopliteal disease and isolated infrapopliteal disease.

Marked disparities were evident in the Primary endpoint events between the rivaroxaban group and the dual-antiplatelet group, as illustrated in Figure 2A. The primary composite efficacy outcome was observed in 108 of the 220 patients (49.1%) on rivaroxaban and in 142 of the 235 patients (39.2%) on dual-antiplatelet therapy (HR 0.70, 95% CI 0.55–0.90, *p* = 0.006), as tabulated in Table 1. Noteworthy differences were discerned between the groups, particularly regarding the escalation of the Rutherford classification—a pivotal metric for lower limb outcomes—and the incidence of target lesion revascularization (TLR). Over a 1-year span, the Rutherford grade augmented by at least one level in 35.5% of patients in the rivaroxaban group compared to 45.1% in the dual-antiplatelet group (HR 0.72, 95% CI 0.54–0.97, *p* = 0.03), depicted in Figure 3. The TLR event incidence at the one-year mark stood at 16.8% for the rivaroxaban group and 27.8% for the dual-antiplatelet group (HR 0.58, 95% CI 0.38–0.86, *p* = 0.007), also portrayed in Figure 3. Other efficacy outcomes exhibited no statistically notable variances between the groups, as detailed in Table 1.

#### Secondary endpoints

Within a span of 1 year, secondary endpoint events manifested in 122 patients (55.5%) in the rivaroxaban cohort and 156 patients (66.4%) in the dual-antiplatelet cohort (HR 0.73, 95% CI 0.58–0.93, *p* = 0.01), as detailed in Table 1. The incidence of pivotal secondary efficacy outcome events, which act as a holistic metric for lower limb outcomes—incorporating factors such as a one-level elevation in Rutherford classification, target vessel recanalization, vascular-driven amputation, and acute limb ischemia—was notably diminished in the rivaroxaban group (45.9%) compared to the dual-antiplatelet group (56.2%) (HR 0.75, 95% CI 0.58–0.97, *p* = 0.03), as showcased in Figure 3. Nevertheless, for other secondary efficacy outcome events, no statistically meaningful disparities were discernible between the two groups, as outlined in Table 1.

#### Safety endpoints

After a year, the primary safety endpoint event, defined as TIMI major bleeding, was recorded in 5.9% of the rivaroxaban cohort and in 2.1% of the dual-antiplatelet cohort (HR 2.6, 95% CI 0.9–7.3, *p* = 0.04), as detailed in Table 3. Notably, other primary safety outcomes, such as intracranial hemorrhage or fatal bleeding, didn't show any significant disparities between the groups. Nonetheless, the secondary safety outcome, characterized by a temporary or permanent discontinuation due to bleeding, exhibited pronounced differences (Table 3). Specifically, by the end of the year, 10% of patients from the rivaroxaban cohort had halted their medication owing to bleeding incidents, in contrast to 4.7% in the dual-antiplatelet cohort (HR 2.2, 95% CI 1.1–4.5, *p* = 0.03) (Table 3).

**Table 3:** Safety outcomes.^a^

Outcome	Rivaroxaban	DAPT	HR (95% CI)	*P*
Principal safety outcome:TIMI major bleeding	13 (5.9)	5 (2.1)	2.6 (0.9, 7.3)	0.04
Intracranial hemorrhage	2 (0.9)	2 (0.9)	1.1 (0.2, 7.6)	0.94
Fatal bleeding	2 (0.9)	3 (1.3)	0.71 (0.12, 4.3)	0.71
Secondary safety outcomes: ISTH major bleeding	17 (7.7)	10 (4.3)	1.9 (0.85, 4.0)	0.11
Any form of bleeding leads to temporary or permanent cessation of medication^b^	22 (10)	11 (4.7)	2.2 (1.1, 4.5)	0.03

^a^ TIMI denotes thrombolysis in myocardial infarction; ISTH denotes International Society on Thrombosis and Haemostasis.

^b^ This category includes adverse events leading to the permanent or temporary discontinuation of a study drug because of a bleeding event that was documented by the investigator on a case­report form; these events included minimal bleeding.

#### Subgroup analyses

The study compared the efficacy of the anticoagulant rivaroxaban plus aspirin and clopidogrel plus aspirin after surgery for above-the-knee and below-the-knee lesions. Comprehensive subgroup baseline characteristics are detailed in Supplementary Materials.

In the subgroup analysis focusing on patients with suprapatellar artery lesions, the rivaroxaban cohort exhibited a statistically significant reduction in both primary and secondary efficacy outcomes compared to the dual-antiplatelet cohort. In stark contrast, for the subgroup with infrapatellar artery lesions, the rivaroxaban cohort did not demonstrate a marked advantage concerning the primary efficacy outcome, as illustrated in Figure 3. Further examination from the subgroup analysis revealed that the primary composite outcome event manifested in 22 patients (28.2%) from the rivaroxaban group and 38 patients (45.2%) from the dual-antiplatelet therapy group, specifically among those with isolated suprapatellar lesions (HR 0.55; 95% CI 0.32–0.92; *P* = 0.02), depicted in Figure 2B.

Conversely, among the infrapatellar lesion-exclusive cohort, the primary composite outcome was noted in 54 patients (58.7%) from the rivaroxaban group and in 59 patients (64.8%) from the dual-antibody group. This difference, represented by an HR of 0.76 (95% CI 0.52–1.09; *P* = 0.14), as shown in Figure 2C, was not statistically significant. For the secondary efficacy composite outcomes in this subgroup, events were observed in 62 patients (66%) from the rivaroxaban group and 65 patients (71.4%) from the dual-antibody group, yielding an HR of 0.79 (95% CI 0.56–1.12; *P* = 0.18), as documented in Table 1. In juxtaposition, within the suprapatellar lesion-exclusive cohort, secondary critical composite events were recorded in 27 patients (35.1%) from the rivaroxaban group and 47 patients (55.3%) from the dual-antibody group, producing an HR of 0.56 (95% CI 0.35–0.90; *P* = 0.02), also tabulated in Table 1.

## Discussion

Our research underscores that the distinct pathological attributes of arterial lesions, whether above or below the knee, wield considerable influence over the post-EVT medication selection. Within the confines of this single-center retrospective investigation, pairing rivaroxaban with aspirin outperformed dual antiplatelet therapy in curtailing adverse limb outcomes associated with lower extremity atherosclerosis. Further granularity, provided by the subgroup analysis, ratified the predominance of rivaroxaban for those with exclusive above-the-knee artery lesions. However, for the isolated below-the-knee artery lesions cohort, the distinctions were not statistically significant. Incorporating a low dosage of rivaroxaban enhanced the prognostic outlook for lower extremity atherosclerosis predominantly impacting the above-the-knee artery.

The arteries both above and below the knee present distinct postoperative pathological features. Investigations into the pathological attributes of vessels in limbs amputated due to chronic limb ischemia reveal a pattern: below-knee arteries predominantly manifest thrombotic lumen occlusion coupled with modest atherosclerosis. In contrast, arteries located above the knee frequently display lumen occlusion resulting from pathological intimal thickening, accompanied by atherofibrosis and fibrous calcified lesions (24, 27). The extent of damage to the vessel wall varies during surgical procedures, including balloon dilation and stent placement, depending on plaque loads and wall pathologies (28, 29). The inherent variability in pathological features across vascular regions might inform the selection of post-EVT therapeutic strategies. Prompted by this potential variability, we delineated two distinct subgroups: those with exclusive above-the-knee lesions and those with isolated below-the-knee lesions. Our intent was to delve deeper into this paradigm by juxtaposing the efficacy of the two therapeutic regimens within these specific cohorts. In the VOYAGER PAD trial, the amalgamation of 2.5 mg of rivaroxaban with 100 mg of aspirin markedly diminished the incidence of major adverse cardiovascular events and significant limb adversities, encompassing primary amputations, when set against aspirin in isolation (17.3% vs. 19.9%, HR, 0.85, 95% CI, 0.76 to 0.96; *P* = 0.009), which is consistent with the findings observed in our study. This therapeutic schema garnered further validation in a comprehensive study spanning over 6,500 patients diagnosed with lower extremity arterial occlusion, affirming its potency (30). In a subgroup analysis of the large-scale clinical study COMPASS, similar examinations were conducted among 6,391 LE-PAD patients. The results indicated that, compared to aspirin monotherapy, the combination of aspirin with a low dose of rivaroxaban reduced peripheral vascular prognosis by 24% (5.5% vs. 7.1%; *p* = 0.03), decreased MALE by 43% (1.5% vs. 2.6%; *p* = 0.01), and reduced amputations by 58% (0.5% vs. 1.2%; *p* = 0.01). These findings align with the positive effects on limb prognosis observed in our study due to a low dose of rivaroxaban (31). While the VOYAGER PAD trial,COMPASS trial and our investigation concur that the combination of rivaroxaban and aspirin post-EVT diminishes the incidence of adverse limb events relative to alternative regimens, they diverge in methodology. Distinctively, our study incorporates a subgroup analysis predicated on the specific vascular territories involved. Through this lens, we discerned that for cases spotlighting isolated below-knee artery lesions, neither therapeutic strategy markedly prevailed in curtailing adverse limb manifestations.

From our subgroup analysis, we can delineate several key insights. Firstly, thrombin predominantly stabilizes formed thrombi rather than instigating their genesis (32). Secondly, platelets play a pivotal role in the onset and escalation of atherosclerotic lesions. Additionally, they are instrumental in the emergence of thrombotic complications, termed atherothrombosis, ensuing from the erosion or rupture of atherosclerotic plaques, manifest in both below-the-knee and above-the-knee arterial territories (33, 34). While thrombin's role in acute arterial thrombosis is secondary to platelets, primarily involved in thrombus stabilization (32). Consequently, postoperative administration of antiplatelet therapy becomes paramount in the management following endovascular treatment (EVT). When one considers the below-the-knee artery—distinguished by its reduced diameter, decelerated blood flow, diminished plaque burden, and infrequent wall disruptions—it's evident that platelet activation predominantly drives thrombus formation (25). Factor Xa, pivotal for thrombin formation, has been linked not only to thrombogenesis but also to inflammation, vascular remodeling, plaque progression, and tissue fibrosis (35, 36). Consequently, inhibiting Factor Xa gains heightened relevance in the context of the above-the-knee artery, where surgically-induced inflammation and pronounced plaque erosion are characteristic (33, 37, 21, 38, 39). Our proposition is that Factor Xa exerts a more pronounced influence on the thrombosis and stabilization within above-the-knee l artery lesions when compared to the less atherogenic below-the-knee artery. In addressing below-the-knee artery lesions, judicious antiplatelet therapy remains paramount. For patients exclusively manifesting below-the-knee artery lesions, a parallel prognosis was discerned under either therapeutic regimen, provided the antiplatelet therapy was optimal. Nevertheless, for post-EVT patients with above-the-knee artery lesions, dual antiplatelet therapy bereft of Factor Xa inhibition was found to be less efficacious than rivaroxaban.

Given the constrained 1-year observational window of this study, there exists the possibility that certain adverse limb events, serving as utility outcomes, might not have been comprehensively documented within this period. In an effort to augment the sensitivity of patient outcome evaluations, an additional tier of the Rutherford classification was integrated into the utility outcome metrics. This nuanced approach facilitated a more discerning assessment of patient prognosis. Instead of exclusively relying on adverse limb events—which primarily function as a diagnostic benchmark for lower extremity arterial disease—the incorporation of an expanded Rutherford classification enhanced the precision of outcome determination (40). Traditionally, research studies have used adverse limb events, such as target lesion revascularization (TLR), acute limb ischemia, and major amputation due to vascular causes, as primary outcomes to assess the efficacy of post-endovascular treatment (EVT) medications on lower limb outcomes (1, 30, 41, 42). In contrast, this study expanded its criteria and did not depend exclusively on the incidence of acute limb ischemia and target lesion revascularization (TLR) as the sole markers of adverse events.

This study identified a higher incidence of TIMI major bleeding, the primary safety endpoint, within the rivaroxaban group (5.9%) as opposed to the dual-antiplatelet group (2.1%) after a year (HR 2.6, 95% CI 0.9–7.3, *p* = 0.04). Other complications, such as intracranial hemorrhage or fatal bleeding, did not show significant differences between the two groups. In terms of ISTH major bleeding, a secondary safety outcome, both groups were comparable. However, a noteworthy finding was that patients in the rivaroxaban group were more likely to discontinue treatment due to bleeding (10% vs. 4.7%, HR 2.2, 95% CI 1.1–4.5, *p* = 0.03). In the VOYAGER PAD trial, there was no significant difference in the incidence of TIMI major bleeding between the rivaroxaban group and the aspirin group (2.65% and 1.87%, respectively; hazard ratio, 1.43; 95% CI, 0.97 to 2.10; *P* = 0.07). However, the incidence of ISTH major bleeding was notably higher in the rivaroxaban group (5.94% vs. 4.06%; hazard ratio, 1.42; 95% CI, 1.10–1.84; *P* = 0.007) (30). While not statistically significant, the bleeding event rate in our study was higher than that in the VOYAGER PAD trial. This discrepancy might be attributed to the dosing in our study, which used 5 mg of rivaroxaban twice daily, whereas the VOYAGER PAD trial used a dose of 2.5 mg twice daily, in addition to the incorporation of clopidogrel in the aspirin group. However, it's pertinent to note that in the COMPASS study, there was no significant difference in bleeding rates between 5 mg twice daily and 2.5 mg twice daily dosing of rivaroxaban. Besides, Below-the-knee arterial lesions are among the significant causes of CLI, and once CLI occurs, the limb salvage rate dramatically decreases (43–46). Given the high rate of adverse limb events associated with below-the-knee arterial lesions, we opted not to alter the rivaroxaban dosage, consistently administering 5 mg twice daily throughout our study. In the COMPASS trial, consistent results were observed. Compared to the aspirin-alone group, the combination of 2.5 mg rivaroxaban with aspirin increased the incidence of major bleeding (77 [3%] out of 2,492 patients vs. 48 [2%] out of 2,504 patients; HR 1.61, 95% CI 1.12–2.31, *p* = 0.0089). Similarly, among 2,474 patients administered 5 mg of rivaroxaban, 79 (3%) experienced major bleeding events, while in the aspirin-alone group of 2,504 patients, 48 (2%) encountered major bleeding (HR 1.68, 95% CI 1.17–2.40; *P* = 0.0043) (32). These evidences indicate a heightened risk of major bleeding and consequent treatment cessation with rivaroxaban. Thus, clinicians must carefully weigh the therapeutic benefits against the potential bleeding risks. Interestingly, despite the elevated bleeding risk, rivaroxaban did not offer a clear advantage over dual-antiplatelet therapy in patients with isolated below-the-knee artery lesions, as evidenced by the subgroup analysis.

## Limitations

Our study has notable methodological constraints. The varied nature of the lesions treated, differences in lesion length, and the use of diverse devices introduce significant variability to our results. Despite acting as a proof-of-concept for post-EVT medications and dosing strategies, our limited sample size restricts comprehensive subgroup analysis. The retrospective approach, even with diligent follow-up, might have missed some events. Considering the high limb event rate for below-the-knee lesions and no clear evidence showing increased bleeding with a higher rivaroxaban dose, we consistently used 5 mg twice daily. However, the lack of a 2.5 mg dose group may hinder optimal dosing conclusions. Additionally, only about 65% of our patients were prescribed statins, possibly affecting atherosclerosis prognosis. This low percentage was due to the region's economic constraints and the high cost of non-insured statins.

## Conclusion

In a retrospective, proof-of-concept study involving patients with symptomatic suprapatellar artery disease undergoing endovascular treatment (EVT), our findings highlighted the effects of specific therapeutic regimens. Specifically, when patients were administered a combination of 5 mg rivaroxaban twice daily alongside standard aspirin, there was a notable reduction in composite adverse outcomes, which encompassed events like elevated Rutherford classification, angioplasty requirements, acute limb ischemia, amputations, revascularizations, myocardial infarctions, strokes, and instances of cardiovascular-related mortality. This therapeutic benefit was particularly prominent in patients exclusively exhibiting suprapatellar artery lesions. Conversely, for those with lesions confined to the below-the-knee artery, the combined regimen of rivaroxaban and aspirin did not surpass the efficacy of dual-antiplatelet therapy. It's crucial to note from a safety standpoint that this combined regimen (5 mg rivaroxaban twice daily with aspirin) was correlated with a heightened risk of major bleeding, resulting in an increased rate of treatment discontinuations when benchmarked against the aspirin and clopidogrel combination.

## Data Availability

The original contributions presented in the study are included in the article/Supplementary Material, further inquiries can be directed to the corresponding author.
