# Supplementary material for: Rivaroxaban plus aspirin vs. dual antiplatelet therapy in endovascular treatment in peripheral artery disease and analysis of medication utilization of different lesioned vascular regions
Source: Front Surg. 2023 Nov 9;10:1285553. doi: 10.3389/fsurg.2023.1285553 (PMC10665835; doi:10.3389/fsurg.2023.1285553)
Supplement: Supplementary file 1 [file Table1.docx]

TableⅠ. Baseline Characteristics of the subgroup of patients with isolated suprapopliteal artery disease

| Variable | Rivaroxaban | DAPT | P |
| --- | --- | --- | --- |
| Median age (IQR) - yr  Female sex-no. (%)  Median body-mass index(IQR)†  Diabetes-no. (%)  Smoking-no. (%)  Current  Former  Never  Alcohol use-no. (%)  Never  Rarely  Currently consumes  Baseline CrCl, mg/dL-no. (%)  ≤50  >50 and <80  ≥80  >95  Hypertension-no. (%)  Cardiovascular disease-no. (%)‡  Carotid artery disease-no. (%)  Cholesterol, mmol/L(IQR)  HDL cholesterol, mmol/L(IQR)  LDL cholesterol, mmol/L(IQR)  Rutherford category-no. (%)  2  3  4  5  Lesion length, cm(IQR)  Lesion severity-no.(%)  Stenosis  Occlusion  Preoperative ABI(IQR)  Postoperative ABI(IQR)  Stent placement-no. (%)  Bare metal  Drug-eluting  Residual stenosis(IQR)  History of vascular angioplasty-no. (%)  Statin-no. (%)  ACE inhibitor or ARB-no. (%) | 76(68.0,81.0)  26（33.3）  25.8(24.5,27.0)  34（43.6）  52(66.7)  0(0)  26(33.3)  37(47.4)  11(14.1)  30(38.5)  5（6.4)  37（47.4）  24（30.7）  12（15.3）  38(48.7)  22(28.2)  22(28.2)  4.3(3.8,5.2)  1.4(0.8,1.6)  2.5(1.5,3.2)  1(1.3)  64(82.1)  10(12.8)  3(3.9)  23.7(21.7,25.6)  26(33.3)  52(66.7)  0.31(0.23,0.38)  0.79(0.70,0.88)  29(37.2)  34(43.6)  12.1(7.1,17.1)  19(24.4)  52(66.7)  49(62.8) | 76(68.0,79.8)  33（39.3）  25.7(25.3,27.4)  42（50.0）  42(50.0)  2(2.4)  40(47.6)  32(38.1)  13(15.5)  39(46.4)  2（2.3）  34（40.4）  37（44.0）  11（13.1）  43(51.2)  24(28.6)  17(20.2)  4.7(4.3,5.3)  1.3(1.0,1.6)  2.4(1.7,3.0)  0(0.0)  63(75.0)  15(17.9)  6(7.1)  24.7(23.1,25.6)  18(21.4)  66(78.6)  0.29(0.16,0.34)  0.78(0.68,0.88)  24(28.6)  33(39.3)  12.7(5.95,19.5)  18(21.4)  65(77.3)  54(64.3) | 0.90  0.43  0.78  0.41  0.05  0.48  0.26  0.75  0.96  0.24  0.14  0.68  0.88  0.43  0.07  0.09  0.16  0.50  0.16  0.57  0.66  0.13  0.85 |

Table II. Baseline Characteristics of the subgroup of patients with isolated infrapopliteal artery disease

| Variable | Rivaroxaban | DAPT | P |
| --- | --- | --- | --- |
| Median age (IQR) - yr  Female sex-no. (%)  Median body-mass index(IQR)†  Diabetes-no. (%)  Smoking-no. (%)  Current  Former  Never  Alcohol use-no. (%)  Never  Rarely  Currently consumes  Baseline CrCl, mg/dL-no. (%)  ≤50  >50 and <80  ≥80  >95  Hypertension-no. (%)  Cardiovascular disease-no. (%)‡  Carotid artery disease-no. (%)  Cholesterol, mmol/L(IQR)  HDL cholesterol, mmol/L(IQR)  LDL cholesterol, mmol/L(IQR)  Rutherford category-no. (%)  2  3  4  5  Lesion length, cm(IQR)  Lesion severity-no.(%)  Stenosis  Occlusion  Preoperative ABI  Postoperative ABI  Balloon placement-no. (%)  Normal  Drug-coated  Residual stenosis(IQR)  History of vascular angioplasty-no. (%)  Statin-no. (%)  ACE inhibitor or ARB-no. (%) | 77(68.0,81.0)  31（33.7）  25.5(22.6,27.8)  43（46.7）  45(48.9)  11(12.0)  36(39.1)  56(50.0)  19(20.7)  27(29.3)  3（3.26)  39（42.4）  31（33.7）  19（20.7）  56(60.9)  28(30.4)  40(43.5)  4.2(3.7,5.1)  1.4(0.8,2.0)  2.6(1.9,3.2)  0(0.0)  41(44.6)  21(22.8)  30(32.6)  12.6(11.4，13.2)  32(34.8)  60(65.2)  0.29(0.21,0.34)  0.77(0.75,0.83)  32(34.8)  60(65.2)  13.0(10.0,16.0)  15(16.3)  54(58.7)  47(51.1) | 78(69.0,82.0)  22（24.2）  25.4(23.1,27.1)  40（44.0）  52(57.1)  13(14.3)  26(28.6)  41(45.1)  26(28.6)  24(26.4)  10（11.0）  38（41.8）  25（27.5）  18（19.8）  60(66.0)  26(28.6)  31(34.1)  4.3(4.0,5.2)  1.4(0.8,2.0)  2.5(1.8,3.2)  0(0.0)  48(52.8)  15(16.5)  28(30.8)  12.7(11.8，13.2)  26(28.6)  65(71.4)  0.27(0.21,0.35)  0.77(0.71,0.84)  34(37.4)  57(62.6)  13.0(7.0,19.0)  19(20.9)  51(56.0)  51(56.0) | 0.58  0.16  0.52  0.71  0.32  0.46  0.22  0.48  0.78  0.19  0.37  0.75  0.41  0.45  0.20  0.37  0.63  0.34  0.72  0.80  0.43  0.72  0.50 |

*There were no significant differences between groups. Percentages may not total 100 because of rounding. Continuous data are presented as the means ± standard deviation（if it matches normal distribution) or Median(interquartile range)(if it matches skewed distribution).; categorical data are given as the counts (percentage). Percentages were based on the number of subjects in the column heading as the denominator unless specified otherwise. Abbreviations: ACE denotes angiotensin-converting enzyme;ARB angiotensin-receptor blocker, ABI, ankle-brachial index;CrCl, creatinine clearance; HDL, high-density lipoprotein; LDL, low-density cholesterol；IQR denotes interquartile range.

†The body-mass index is the weight in kilograms divided by the square of the height in meters.

‡Cardiovascular diseaseis is defined as myocardial infarction, percutaneous coronary intervention, or coronary­artery bypass grafting (CABG).
